# GLD-IPOR Malawi COVID-19 panel survey dataset

**DOI:** 10.1016/j.dib.2025.111485

**Published:** 2025-03-17

**Authors:** Erica Ann Metheney, Victor Saidi Phiri, Samuel Tafesse Wakuma

**Affiliations:** University of Gothenburg, 405 30 Gothenburg, Sweden

**Keywords:** Pandemic response, Restrictions, Authority, Elections, Community, Stigma, Malawi

## Abstract

March 2020 marked a critical juncture for Malawi as the nation confirmed its initial COVID-19 cases. In response, the government imposed stringent measures including travel restrictions, bans on large gatherings, and the creation of emergency management committees to mitigate the spread of the virus. Concurrently, Malawi navigated a significant political event—the June 2020 presidential election—following the annulment of the previous year's election results.

To document the range of responses by Malawian citizens to these COVID-19 containment strategies and how their livelihoods and political engagement were affected, a three-wave survey was conducted. Captured in the GLD-IPOR Malawi COVID-19 Panel Survey Dataset, this effort provides detailed insights into public knowledge and perceptions of COVID-19, socio-economic and health vulnerabilities prompted by the pandemic, citizens' adherence to public health directives, and engagement during this crisis. Notably, the survey waves were aligned with the June 23, 2020, presidential electoral cycle: wave one occurred during the pre-election period, and wave two immediately followed the election. The dataset, which includes 13,696 observations collected over all three rounds offers a comprehensive understanding of local variation in responses – particularly in terms of social stigmatization, enforcement of containment measures, and political participation – during a period marked by both public health challenges and political transitions.

This dataset can be leveraged to offer actionable insights and opportunities for examining resilience and vulnerabilities during different stages of health crises. Furthermore, merging this dataset with the Local Governance Performance Index (LGPI) survey datasets from 2016 and 2019, or the forthcoming project ``Survive, Thrive, or Deprive? Drivers and Outcomes of Resilience During the COVID-19,'' can facilitate a detailed examination of governance and development issues before, during, and after the pandemic.

Specifications Table

**Table:** 

Subject	Social Science
Specific subject area	Local attitudes and responses to the COVID-19 pandemic and 2020 presidential election in Malawi
Type of data	Tables (summary statistics), Survey Data (.rds, .dta, .tab)
Data collection	The telephone survey consisted of three rounds fielded between May 2020 and May 2021. The sampling frame was comprised of respondents from the Local Governance Process Indicators (LGPI) 2019 household survey [1] from Central and Northern Malawi, plus respondents from the LGPI 2016 survey [2] from the Southern region. Only those agreeing to follow-up contact and whose phone numbers were available were included in the sampling frame. Respondents were telephoned, and attempts were made to match them to previous rounds using survey demographics. If unmatched, the call recipient was invited to participate. The sampling frame for successive rounds thus includes previous and new participants, excluding refusals. Responses were recorded using the SurveyToGo app from Dooblo.
Data source location	Country: Malawi Regions: Northern, Central, Southern
Data accessibility	The survey data, codebook, survey questionnaires, sampling information, and the merging keys are available via the Harvard Dataverse. Repository name: Harvard Dataverse Data identification number: 10.7910/DVN/MOOI8X Direct URL to data: https://doi.org/10.7910/DVN/MOOI8X
Related research article	None

## 1 Value of the Data

• This data offers comprehensive information on citizens’ knowledge, fears, and local variations in response practice during the COVID-19 pandemic as well as information on the impact of the pandemic on livelihoods across economic, social, and political dimensions. Additionally, it provides unique data about an election during a public health crisis.
• This data can generate information and actionable insights for government officials, civil society organizations, donors, and other stakeholders developing strategies to mitigate the social, economic, and political implications of pandemics.
• This data provides an opportunity for academics to study the complex relationships between vulnerabilities and resilience at the individual, household, and community levels during times of crisis. Also, the inclusion of a range of community attitudes and responses over time provides a useful framework for similar studies.
• Merging this dataset with the LGPI 2019 or LGPI 2016 surveys — which are administered through face-to-face household interviews and gather detailed micro-level insights into citizens’ experiences, perceptions, and satisfaction with governance processes across a broad range of topics in various sectors—allows for exploration of the relationship between pre-pandemic and pandemic-era conditions, as well as an analysis of how the pandemic has impacted the livelihoods in Malawi. These surveys not only enable a retrospective study but also lay the groundwork for future crisis research.
• The alignment of the presidential election period with the concurrent health crisis allows for an analysis of election dynamics during the pandemic. By capturing citizens' political perceptions and participation in Round 1 and Round 2 of the survey, the dataset provides insights, including but not limited to, how fear of infection and economic distress may have influenced trust in government, willingness to vote, and electoral choices during a public health crisis.
• When merged and/or compared with the data obtained from the Governance and Local Development's forthcoming project, “Survive, Thrive, or Deprive? Drivers and Outcomes of Resilience During the COVID-19” which is funded by the Swedish Research Council, it will allow for the analysis of questions about experiences among communities that span the pre-, peri- and post-pandemic periods.

## 2 Background

Following a February 2, 2020, court ruling that mandated a new presidential election, Malawi entered the pandemic era in March 2020 with its first reported COVID-19 cases. This led to the implementation of preventive measures, including travel restrictions and bans on public gatherings. By the time Malawians cast their ballots on June 23, 2020, the nation was grappling with both a health emergency and a political transition. This unprecedented confluence of events not only introduced significant socioeconomic challenges directly related to the pandemic but also had the potential to influence citizens’ political perceptions, participation, and ultimately shape the election outcome. Leveraging our existing respondent pool from the Local Governance Performance Index (LGPI) surveys of 2016 and 2019, who had consented to be recontacted, we embarked on a broader effort to document local responses to the COVID-19 pandemic over time and the challenges faced during this peculiar political transition period, through three survey rounds in 2020.

These surveys aimed to investigate various facets such as awareness of COVID-19, attitudes towards its health and economic impacts, vulnerabilities, and compliance with preventive measures. Emphasis was placed on examining the local differences in stigma, enforcement measures, interaction with authorities, and the pandemic dynamics with citizens’ political participation.

Our overarching objective was to create a foundational resource for understanding local responses to the pandemic. By gathering data on citizens' knowledge, attitudes, and behaviors regarding COVID-19 containment measures, our study aimed to enrich the theoretical and methodological understanding of how different communities react to health emergencies. This provides valuable insights into pandemic management and fosters further exploration of responses to health emergencies.

## 3 Data Description

The GLD-IPOR Malawi COVID-19 Panel Survey Dataset is housed in the Harvard Dataverse repository. The data itself is located in the ``data'' folder, while the merging keys and documentation can be found in the ``documentation'' folder. Additionally, we have a document that outlines the process for merging the rounds of the COVID survey with the LGPI datasets from 2016 or 2019.

The survey data was collected in three rounds during the COVID-19 pandemic in Malawi. The first round was collected from May 7 – June 2, 2020 (n = 4658), the second round from August 21 – October 7, 2020 (n = 4908), and the third round from March 9 - May 1, 2021 (n = 4127). Trained enumerators administered the surveys by phone and recorded responses on tablets or computers.

The surveys occurred at different stages of the COVID-19 pandemic, from very early when cases were low, to advanced stages when the disease had spread extensively. The rounds also occurred at various points around the 2020 presidential election. This changing dynamic, as well as the results from previous rounds, informed changes in questions from round to round. Table 1 charts the different topics investigated in each round of the survey.

**Table 1:** Topics covered in the different survey rounds available in the dataset.

Topic	Round 1	Round 2	Round 3
**Demographics**			
Respondent Demographics	x	x	x
Pre-Existing Health Condition	x	x	x
**COVID-19-related information**			
Knowledge of COVID-19 and Symptoms	x		
Have/Had COVID Symptoms	x	x	x
Sources of COVID-19 Information	x		
Moved because of COVID			x
Tested for COVID		x	x
Attitudes Towards COVID-19 Testing	x	x	x
Opinion on Future severity		x	x
COVID Stigma		x	x
Change in Daily life		x	x
Precautionary Behaviors	x	x	x
Use of Healthcare	x	x	x
Community Restrictions		x	x
Economic Impacts and Fears	x	x	x
Hunger Impact and Fears	x	x	x
Health Impact and Fears	x	x	
Crime Impact and Fear		x	x
Social Assistance	x		
Assistance Need and Relief	x	x	x
Access to Soap, Sanitizer, and Water	x	x	x
**Presidential election-related information**			
Existence of Influential People			x
Perceptions of Government	x	x	x
Political Opinion and Affiliations	x	x	x
Election Assessment and Sentiment	x	x	

The respondents cover all three regions (North, Central, Southern) in Malawi, but do not form a representative sample of those regions. Table 2 shows distribution of the respondents’ gender, location, and age.

**Table 2:** Respondent characteristics by survey round.

	Round 1	Round 2	Round 3	National Statistics [3]
**Total**	**4658**	**4908**	**4127**	**17563749**
**Gender**				
Men	44 %	44 %	49 %	49 %
Women	56 %	56 %	51 %	51 %
**District**				
Balaka	3 %	3 %	3 %	2 %
Blantyre	9 %	9 %	8 %	3 %
Chikwawa	7 %	8 %	8 %	3 %
Chiradzulu	<1 %	<1 %	<1 %	2 %
Chitipa	2 %	2 %	3 %	1 %
Dedza	5 %	10 %	10 %	5 %
Dowa	8 %	8 %	7 %	4 %
Karonga	3 %	3 %	4 %	2 %
Kasungu	9 %	8 %	8 %	5 %
Likoma	<1 %	NA	<1 %	<1 %
Lilongwe	6 %	4 %	5 %	9 %
Machinga	<1 %	<1 %	<1 %	4 %
Mangochi	5 %	4 %	4 %	7 %
Mchinji	4 %	5 %	3 %	3 %
Mulanje	5 %	5 %	5 %	4 %
Mwanza	<1 %	NA	1 %	1 %
Mzimba	10 %	10 %	8 %	5 %
Neno	<1 %	NA	NA	1 %
Nkhatabay	3 %	<1 %	2 %	2 %
Nkhotakota	5 %	4 %	4 %	2 %
Nsanje	4 %	4 %	4 %	2 %
Ntcheu	5 %	5 %	6 %	4 %
Ntchisi	<1 %	<1 %	<1 %	2 %
Phalombe	<1 %	<1 %	<1 %	2 %
Rumphi	3 %	2 %	3 %	1 %
Salima	<1 %	<1 %	<1 %	3 %
Thyolo	<1 %	<1 %	<1 %	4 %
Zomba	5 %	5 %	4 %	4 %
**Age**				
18 - 25	23 %	21 %	19 %	16 %
26 - 45	53 %	55 %	56 %	22 %
46 - 65	18 %	19 %	20 %	8 %
Older than 65	5 %	5 %	5 %	3 %

Using questions early in the survey, respondents were classified as either previous participants or new participants. If an enumerator was unsure, the respondent was classified as new. This information was used to determine which survey rounds each respondent participated in. The distribution of respondents by survey round participation is shown in Table 3.

**Table 3:** Proportion of survey respondents across rounds.

Survey Round	Frequency	Percent
LGPI2016 + MCSR1	260	3 %
LGPI2016 + MCSR1 + MCSR2	459	5 %
LGPI2016 + MCSR1 + MCSR2 + MCSR3	519	6 %
LGPI2016 + MCSR1 + MCSR3	64	<1 %
LGPI2016 + MCSR2	565	6 %
LGPI2016 + MCSR2 + MCSR3	487	6 %
LGPI2016 + MCSR3	236	3 %
LGPI2019 + MCSR1	387	4 %
LGPI2019 + MCSR1 + MCSR2	712	8 %
LGPI2019 + MCSR1 + MCSR2 + MCSR3	525	6 %
LGPI2019 + MCSR1 + MCSR3	138	2 %
LGPI2019 + MCSR2	367	4 %
LGPI2019 + MCSR2 + MCSR3	339	4 %
LGPI2019 + MCSR3	310	4 %
MCSR1	1319	15 %
MCSR1 + MCSR2	133	2 %
MCSR1 + MCSR2 + MCSR3	105	1 %
MCSR1 + MCSR3	37	<1 %
MCSR2	374	4 %
MCSR2 + MCSR3	323	4 %
MCSR3	1044	12 %

Accompanying the dataset, we also provide a comprehensive codebook. This codebook includes detailed information such as variable names, full question text, response rates, distribution of answers, and sampling information.

## 4 Experimental Design, Materials and Methods

The present dataset, collected from a sample of Malawians between May 7, 2020, and May 1, 2021, involved various scholars from all over the world, including country topic experts. The survey instrument used to collect the data employs randomization, jump rules, and survey logic to maintain respondent engagement and minimize fatigue. Filtering capability and enumerator instructions are also embedded, enabling the verification of previous round respondents when needed and ensuring seamless navigation through the questions. Depending on the type and detail of information needed for a specific section of the survey, different types of questions ranging from a simple single choice to an open-ended format were included. We have also embedded experimental elements in these surveys which we have not included in the datasets. To ensure full transparency regarding the survey's structure, the entire questionnaire for each round is available in English on the Harvard Dataverse. The questionnaire items are categorized into chapter topics, with experimental items clearly identifiable within their respective chapter.

The initial sampling frame for the first round of the panel consisted of 9,140 contacts of which 5,014 were previous LGPI 2019 respondents and 4,126 associated with LGPI 2016 survey (not necessary respondents of the survey). The LGPI 2019 survey was only conducted in the northern and central districts and was not nationally representative. To incorporate the southern districts, enumerator teams were sent, very early in the pandemic, to the southern and southern-central region villages that had been covered in the LGPI 2016 survey. Enumerators were given and instructed to wear masks, use hand sanitizer, and maintain social distancing measures. These enumerators met with village heads who sent a village member to contact previous respondents and collect phone numbers from those who were willing to participate. Where an individual was either unwilling or unavailable, another adult in the household was asked to participate. If no one from the original household agreed to be contacted, another household in the village willing to be contacted was approached.

We used name, time lived in the area, gender, age, and education to verify whether the individual was the same respondent contacted in previous surveys or recruited from the southern region. If the respondent was not available but the individual answering the phone was over 18, the individual was asked if they wanted to participate in the study. If the individual answering the phone was under 18 and the initial respondent was not available, the enumerator asked if an adult was available. The adult was then given the choice to participate in the survey.

The sampling frame utilized in Round 2 and Round 3 of the COVID Survey was an extension of the sampling frame employed in the preceding rounds. Thus, the sampling frame for successive rounds includes respondents from the previous survey's sampling, plus any new respondents, and less any respondents that refused further contact.

The survey was coded using the software SurveyToGo Studio and implemented using the SurveyToGo app from Dooblo. The surveys were implemented by the Institute of Public Opinion and Research (IPOR) in Malawi. Daily monitoring reports and weekly enumerator reports were generated during survey fielding to catch survey errors, poor enumerator performance, and improve overall data quality.

## Limitations

• Sampling Frame: The data is not nationally representative or representative of the regions.
• Attrition: The three rounds of surveys do not have an equal number of observations due to the high number of respondents who were not willing to participate. As such, we have an unbalanced panel.
• Phone-based Survey Challenges: While telephone surveys were a safe way to collect data during the COVID-19 pandemic, according to data from the International Telecommunication Union, in 2019, there were approximately 47 mobile cellular subscriptions per 100 people in Malawi [4]. This could result in an under-representation of lower income or rural based individuals, an issue common to telephone surveys. However, our sampling frame was derived from respondents to a face-to-face survey that utilized a sampling frame that intentionally included respondents from rural and hard to reach areas. Thus, our sample is more likely to include individuals with access to cell phones in lower income and/or rural areas than one would expect via other methods such as random digit dialing. Additionally, the callback procedure for the panel surveys utilized a high number of recall attempts, and longer callback intervals to account for intermittent charging, network disruptions, and phone sharing practices.

## Ethics Statement

This study received in-country ethics and regulatory approval from the University of Malawi (P.05/20/17 (2020-06-17)). We note that the study did not receive approval from the researchers’ home institution/country because the Swedish Ethnical Review Authority will not provide IRB approval for research conducted outside of Sweden. The project leaders were trained in courses on the ethical treatment of human research participants.

This study was conducted during the COVID-19 pandemic, and as a result special safety measures were taken. When enumerators were collecting phone numbers from LGPI 2016 survey participants, there were instructed to wear masks, use hand sanitizers, and maintain social distancing measures. Additionally, three measures were taken to reduce face-to-face interactions during fielding: (1) the survey was administered via telephone, (2) enumerator training was hosted online, (3) enumerators were able to administer surveys from home.

All handling and storage of personal data was performed in accordance with the General Data Protection Regulation (GDPR). Enumerators were given password protected tablets and assigned unique login credentials for the SurveyToGo app that they used to collect data from respondents. After a session was complete, enumerators uploaded the observations to the SurveyToGo server, removing the data from their tablets. The SurveyToGo server is located in the United States and owned by Dooblo which is ISO 27001 certified for information security and management.

Access to the raw survey data on the SurveyToGo server is restricted to the Principal Investigators and Data Managers through personal login credentials. The raw and processed versions of the survey dataset that contain personal data are stored at the University of Gothenburg in the Governance and Local Development Institute's secure data storage which has been certified to securely store data up to Class III. The server for this storage is located in a locked and alarmed room and the data is backed up every two weeks. Access to the secure storage location at the University of Gothenburg is restricted to the Data Managers, accessibly only through dual authentication.

Informed consent was received for each survey and only respondents over 18 years of age were allowed to participate. To mitigate respondent discomfort, every question included a Don't Know/Refuse to Answer option. After the end of each survey, the respondent was asked to consent to follow up contact for the subsequent round. Respondents that declined were removed from the sampling frame. Additionally, participants were financially compensated for the participation in airtime for in all rounds, although this was not explicitly stated in the Round 1 consent statement.

**Round 1**

We are currently conducting a survey on COVID-19 and would like to talk to you today. Your answers will be confidential. They will be put together with about 3500 other people we are talking to, to get an overall picture. It will be impossible to pick you out from what you say, so please feel free to tell us what you think. This interview will take about 15 min. There is no penalty for refusing to participate.

We would like your opinion with the knowledge that there are no right or wrong answers to these questions and that you may stop the survey at any time.

Are you willing to participate in this survey, either now or at another time?

**Round 2**

Participation in our COVID-19 survey is voluntary, and your answers will be confidential. They will be put together with about 3500 other people we are talking to, to get an overall picture. It will be impossible to pick you out from what you say, so please feel free to tell us what you think. This interview will take about 30 min. There is no penalty for refusing to participate.

We would like your opinion with the knowledge that there are no right or wrong answers to these questions and that you may ask for clarification or stop the survey at any time. You are also free to skip questions you consider personal or invasive without penalty.

We are able to offer you 1000 Kwacha in airtime for completing the survey.

Are you willing to participate in this survey, either now or at another time?

**Round 3**

Participation in our COVID-19 survey is voluntary, and your answers will be confidential. They will be put together with about 4000 other people we are talking to, to get an overall picture. It will be impossible to pick you out from what you say, so please feel free to tell us what you think. This interview will take about 30 min. There is no penalty for refusing to participate. We would like your opinion with the knowledge that there are no right or wrong answers to these questions and that you may ask for clarification or stop the survey at any time. You are also free to skip questions you consider personal or invasive without penalty.

By agreeing to take the survey, you are giving us the right to transfer the information you provide to our research partners at the Program on Governance and Local Development at the University of Gothenburg in Sweden.

You may withdraw your consent for us to process your data at any time by calling [PHONE NUMBER WE CHOSE TO USE].

We are able to offer you 500 Kwacha in airtime for completing the survey.

Are you willing to participate in this survey, either now or at another time?

## CRediT authorship contribution statement

**Erica Ann Metheney:** Data curation, Writing – review & editing. **Victor Saidi Phiri:** Data curation, Writing – original draft. **Samuel Tafesse Wakuma:** Data curation, Writing – original draft.

## Data Availability

DataverseGLD-IPOR Malawi Covid Panel Survey (Original data).
